# Systematic Survey of *Vibrio* spp. and *Salmonella* spp. in Bivalve Shellfish in Apulia Region (Italy): Prevalence and Antimicrobial Resistance

**DOI:** 10.3390/microorganisms11020450

**Published:** 2023-02-10

**Authors:** Maria Emanuela Mancini, Alessandra Alessiani, Adelia Donatiello, Antonella Didonna, Luigi D’Attoli, Simona Faleo, Gilda Occhiochiuso, Francesco Carella, Pietro Di Taranto, Lorenzo Pace, Valeria Rondinone, Annita Maria Damato, Rosa Coppola, Carmine Pedarra, Elisa Goffredo

**Affiliations:** Istituto Zooprofilattico Sperimentale della Puglia e della Basilicata, Via Manfredonia 20, 71121 Foggia, Italy

**Keywords:** antibiotic resistance, broth microdilution, marine environment, surveillance, foodborne pathogens

## Abstract

The emergence of antimicrobial resistance (AMR) is increasingly common across the globe and aquatic ecosystems could be considered a reservoir of antibiotic-resistant bacteria. This study aimed to determine prevalence and antibiotic susceptibility of the potential pathogenic bacteria *Salmonella* spp. and *Vibrio* spp. in bivalve molluscs intended for human consumption, collected over a period of 19 months along the northern coast of Apulia region. The AMR profile was also determined in non-pathogenic *Vibrio* species, common natural inhabitants of seawater and a useful indicator for the surveillance of AMR in the environment. The current study presents data on the AMR of 5 *Salmonella* and 126 *Vibrio* isolates by broth microdilution MIC. Multidrug resistance (MDR) was observed in one S. Typhimurium strain towards sulfamethoxazole, trimethoprim, tetracycline, gentamicin, and ampicillin and in 41.3% of the *Vibrio* strains, mostly towards sulphonamides, penicillin, and cephems. All *Vibrio* isolates were sensitive to azithromycin, chloramphenicol, tetracycline, amoxicillin/clavulanic acid, gentamicin, streptomycin, amikacin, and levofloxacin. The AMR phenomenon in the investigated area is not highly worrying but not entirely negligible; therefore, in-depth continuous monitoring is suggested. Results concerning the antibiotic agents without available specific clinical breakpoints could be useful to upgrade the MIC distribution for *Vibrio* spp. but, also, the establishment of interpretative criteria for environmental species is necessary to obtain a more complete view of this issue.

## 1. Introduction

The global increasingly spreading antimicrobial resistance (AMR) is such an alarming current issue that many scientific activities have been implemented worldwide to analyse the phenomenon and to conceive effective contrast measures. Several bacterial species are involved in this phenomenon and the zoonotic ones are clearly of greater concern. Resistant bacteria can affect humans directly through consumption of contaminated food or indirectly by transferring mobile genetic elements (e.g., plasmids, transposable elements, super-integron, and integrating conjugative elements genes) for antibiotic resistance to human pathogens [1,2]. Antibiotic-resistant bacteria (ARB) have emerged in the marine environment as a consequence of the excessive use of antibiotics in human, agriculture, and aquaculture systems during the past few decades [3]. Despite the use of antimicrobials in aquaculture being considered a leading cause of development of ARB [1], only five active substances are approved and registered in Italy: amoxicillin, flumequine, oxytetracycline, chlortetracycline, and sulfadiazine-trimethoprim. 

*Vibrio* species could represent an important indicator of the presence of antibiotic resistance in the marine and estuarine ecosystems, as they are natural inhabitants of coastal waters. Approximately 12 *Vibrio* spp. can cause infections in humans via oral route by ingestion of contaminated water or raw or undercooked contaminated seafood (in particular, *Vibrio parahaemolyticus* and *V. cholerae*, but also *V. vulnificus*, *V. fluvialis*, *V. mimicus*, and *V. hollisae*) or through skin wound exposure to contaminated water, which could result in secondary septicaemia (especially *V. vulnificus* and, to a lesser extent, *V. alginolyticus*) [4]. Shellfish can easily harbour pathogenic microorganisms because of their filter-feeding behaviour and *Vibrio* have been proposed as the most common bacteria responsible for food poisoning after shellfish consumption [2]. *V. cholerae* is the causative agent of cholera, a severe worldwide disease affecting mainly children <5 years of age and with frequent cases of deaths. *V. cholerae* strains are classified into more than 200 serogroups on the basis of the chemical composition of the O antigen of lipopolysaccharide (LPS): strains belonging to serogroup O1 and O139 Bengal are responsible for the vast majority of cholera cases, while non-O1 and non-O139 strains can cause sporadic gastrointestinal and extraintestinal infections [4]. The detection of virulence-associated factors is useful to discriminate between pathogenic and non-pathogenic strains: the *stn/sto* genes encoding the heat-stable enterotoxin are one of the main factors associated with enteropathogenicity in *V. cholerae* [5]. *V. parahaemolyticus* is the most common pathogen causing seafood-borne illnesses in many countries by eating raw or undercooked shellfish, and the strains harbouring the *tdh* (encoding thermostable direct hemolysin) and *trh* (encoding tdh-related hemolysin) genes are pathogenic in humans [6,7]; moreover, it is able to form biofilm on mussel surface [8]. V. *vulnificus* is usually considered an opportunistic pathogen affecting mostly immunocompromised individuals, but it is a highly fatal human pathogen, responsible for >95% of seafood-related deaths in the United States. *Vibrio alginolyticus* causes mainly superficial wound and ear infections, generally cured by an appropriate antibiotic therapy, although rare cases of septicaemia may also occur [4]. However, this species is widespread in the marine environment; therefore, it may represent an essential indicator of environmental antibiotic resistance more than others. *V. harveyi* is one of the most common species infecting farmed aquatic animals [9,10]; however, sporadic cases of human infections, especially wound infections, have been reported in recent years, also, in the Mediterranean Sea [11,12]. *Vibrio* spp. is one of the zoonotic agents listed in Annex I to Directive 2003/99/EC [13] to be monitored according to the epidemiological situation. In Italy, vibriosis outbreaks occurred and occur nowadays, as recently reported in the European Union One Health Zoonoses Report 2019 [14], and, also, the Apulia region was historically involved in important cholera epidemics in the past years [15,16]. Therefore, research activities and monitoring of *Vibrio* spp. are of great interest in the Apulian territory. 

*Salmonella* spp. is another important zoonotic pathogen frequently found in bivalve molluscs and for which the occurrence of multidrug resistance is widely reported also in the areas investigated in the present work [17]. Moreover, *Salmonella* spp. is one of the bacteria for which the monitoring and reporting of AMR is mandatory and specific guidelines on the antimicrobial susceptibility testing (AST) have been laid down [13,18,19]. 

In 2015, Member States adopted the Global Action Plan on AMR and Italy issued the National Action Plan on AMR 2022–2025 in September 2022. Following the principles announced in the Global Action Plan on AMR, it is necessary to have a consistent, standardised approach to collecting and reporting resistance data, so that trends and patterns of resistance evaluated at national, regional, and local level should guide targeted policy decisions to contrast the phenomenon [20].

Starting from these assumptions, the present study aimed primarily to investigate the presence of potential pathogenic bacteria belonging to the genera *Salmonella* and *Vibrio* in edible bivalve molluscs collected systematically along the northern Apulian coast for subsequent evaluation of the AMR profile of isolates, since antimicrobial resistance surveillance in the marine environment is a crucial aspect to implement effective local antibiotic reduction programs. 

## 2. Materials and Methods

### 2.1. Sampling

Overall, 296 shellfish samples were collected from March 2021 to October 2022 along the northern Apulian coast (provinces of Foggia and Barletta-Andria-Trani). Most of them (263/296; 88.85%) derived from the implementation of the official classification programme of bivalve mollusc production and harvesting areas with regard to the Commission Implementing Regulation 2019/627 [21]. The geographical distribution of sampling points was established after a sanitary survey in order to choose the location at highest risk of faecal pollution and ensure that analytical results were representative of the area. At least 12 samples were taken from each sampling point over at least a 6-month period, as recommended in the community guide to the principles of good practice for the microbiological classification and monitoring of bivalve mollusc production and relaying areas with regard to Implementing Regulation 2019/627 [22]. The interval between two sampling occasions was approximatively 2 weeks, depending on the weather conditions and the availability of a commercial-size product. It is important to highlight that search for *Salmonella* is mandatory according to the European legislation [23]; otherwise, *Vibrio* detection was carried out only for research purposes. 

The remaining samples (33/296; 11.15%) consisted of live bivalve molluscs originated from the same investigated areas and sampled for official control in accordance with the Integrated Regional Control Plan of Apulia region; they were collected in purification and dispatch centres or at retail and also tested for the aim of this study.

**Figure 1:**
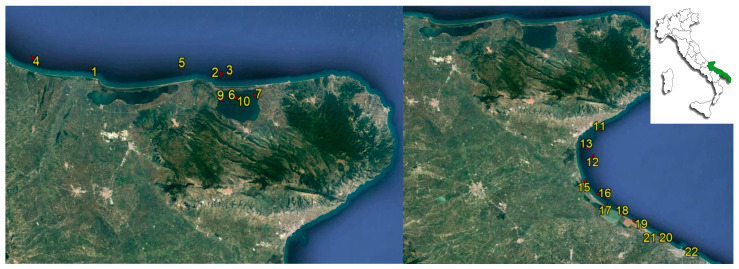
Sampling station locations.

As regards the specimens collected for the official classification purpose, they consisted of oysters (*Crassostrea gigas*), mussels (*Mytilus galloprovinciallis* and *Modiolus barbatus*), clams (*Venus gallina/Chamelea gallina*), cockles (*Acanthocardia tuberculata*), and Japanese carpet shells (*Ruditapes philippinarum*). They were collected from fixed sampling stations, transported to the laboratory on the same day at temperatures between 4 and 10 °C and processed within 24 h of arrival. Environmental parameters, such as water temperature (°C) and pH, were measured on site during the sampling. The sampling stations are illustrated in Figure 1. 

Detailed information about mollusc species and sampling points are reported in Table 1.

### 2.2. Vibrio Detection and Antimicrobial Susceptibility Testing

*Vibrio* spp. detection was performed according to the standard ISO 21872–1:2017 [24] by preparing two enrichment broths, which were incubated at 37 ± 1 °C and 41.5 ± 1 °C, respectively, to enhance the recovery of most *Vibrio* species. All the presumptive *Vibrio* spp. isolates were identified at species level based on the API^®^ ID 20E and 20NE systems (BioMérieux, Nürtingen, Germany), the halotolerance test with various concentrations of NaCl (0, 6 and 10%), and the MALDI-TOF MS (Bruker Daltonics, Bremen, Germany) procedure by the direct transfer method, as previously described [25]. Moreover, the isolates recognised as *V. parahaemolyticus*, *V. cholerae*, and *V. vulnificus* were confirmed by the conventional PCR method described in Annex C to ISO 21872–1:2017, which considers, also, the detection of *V. parahaemolyticus* virulence genes (*tdh* and *trh*).

The broth microdilution MIC method was carried out on colonies grown on nonselective medium saline nutrient agar with 1% NaCl (SNA) incubated at 36 ± 1 °C overnight, using Sensititre™ Gram Negative GN4F^®^ AST Plate and Sensititre™ NARMS^®^ Gram Negative CMV4AGNF AST Plate (Thermofisher Scientific, Paisley, UK). Sensititre™ Gram Negative GN4F^®^ AST Plate contained the following antimicrobials: amikacin (AMI 8–32 µg/mL), ampicillin (AMP 8–16 µg/mL), ampicillin/sulbactam (A/S2 4/2–16/8 µg/mL), aztreonam (AZT 1–16 µg/mL), cefazolin (FAZ 1–16 µg/mL), cefepime (FEP 4–32 µg/mL), ceftazidime (TAZ 1–16 µg/mL), ceftriaxone (AXO 0.5–32 µg/mL), ciprofloxacin (CIP 0.5–2 µg/mL), doripenem (DOR 0.5–4 µg/mL), ertapenem (ETP 0.25–8 µg/mL), gentamicin (GEN 2–8 µg/mL), imipenem (IMI 0.5–8 µg/mL), levofloxacin (LEVO 1–8 µg/mL), meropenem (MERO 0.5–8 µg/mL), minocycline (MIN 1–8 µg/mL), nitrofurantoin (NIT 32–64 µg/mL), piperacillin (PIP 16–64 µg/mL), piperacillin/tazobactam constant 4 (P/T4 8/4–128/4 µg/mL), tetracycline (TET 4–8 µg/mL), ticarcillin/clavulanic acid constant 2 (TIM2 8/2–64/2 µg/mL), tigecycline (TGC 1–8 µg/mL), tobramycin (TOB 2–8 µg/mL), and trimethoprim/sulfamethoxazole (SXT 2/38–4/76 µg/mL). Sensititre™ NARMS^®^ Gram Negative CMV4AGNF AST contained the following antimicrobials: amoxicillin/clavulanic acid 2:1 ratio (AUG2 1/0.5–32/16 µg/mL), ampicillin (AMP 1–32 µg/mL), azithromycin (AZI 0.25–32 µg/mL), cefoxitin (FOX 0.5–32 µg/mL), ceftriaxone (AXO 0.25–64 µg/mL), chloramphenicol (CHL 2–32 µg/mL), ciprofloxacin (CIP 0.015–4 µg/mL), gentamicin (GEN 0.25–16 µg/mL), meropenem (MERO 0.06–4 µg/mL), nalidixic acid (NAL 0.5–32 µg/mL), streptomycin (STR 2–64 µg/mL), sulfisoxazole (FIS 16–256 µg/mL), tetracycline (TET 4–32 µg/mL), and trimethoprim/sulfamethoxazole (SXT 0.12/2.38–4/76 µg/mL). The antibiotics used in the study were chosen in accordance with Clinical and Laboratory Standards Institute (CLSI) recommendations, including those for the treatment of *Vibrio* infections. It was decided to use these Sensititre™ plates in order to include most of the antibiotic compounds listed in CLSI M45 guidelines [26], even if some molecules were present in both antimicrobial plates but at different concentrations. 

The bacterial inoculum was prepared by dissolving a fresh pure colony in 2.5% NaCl solution using a sterile cotton swab until it achieved the turbidity of the 0.5 McFarland standard. Then, 0.1 mL of this suspension was added to 9.9 mL of cation-adjusted Mueller–Hinton broth (Becton Dickinson, Milan, Italy), the antimicrobial plate wells were inoculated with 50 µL of this suspension, and the plate was incubated aerobically at 36 ± 1 °C for 24 h. *Escherichia coli* ATCC^®^ 25922 was used as quality control in each batch. The results were interpreted according to CLSI clinical breakpoints specific for *Vibrio* spp. No CLSI breakpoints were available for the following agents: ceftriaxone, nalidixic acid, streptomycin, tigecycline, ticarcillin/clavulanic acid, nitrofurantoin, doripenem, minocycline, ertapenem, tobramycin, and aztreonam. Hence, results of broth microdilution assays referring to the above-mentioned molecules are reported in the present study without any interpretative criteria. Only for streptomycin, the breakpoints described by other authors were used [27,28].

### 2.3. Salmonella Detection and Antimicrobial Susceptibility Testing

*Salmonella* spp. detection and serotyping were performed according to the ISO 6579–1:2017/Amd 1:2020 [29] and ISO/TR 6579–3:2014 [30], respectively. Antimicrobial susceptibility testing was performed on *Salmonella* strains by broth microdilution using Sensititre™ EUVSEC3^®^ (Termofisher Scientific, Paisley, UK), which contained the compounds specified in the Commission Implementing Decision (EU) 2020/1729: ampicillin (1–32 µg/mL), azithromycin (2–64 µg/mL), amikacin (4–128 µg/mL), gentamicin (0.5–16 µg/mL), tigecycline (0.25–8 µg/mL), ceftazidime (0.25–8 µg/mL), cefotaxime (0.25–4 µg/mL), colistin (1–16 µg/mL), nalidixic acid (4–64 µg/mL), tetracycline (2–32 µg/mL), trimethoprim (0.25–16 µg/mL), sulfamethoxazole (8–512 µg/mL), chloramphenicol (8–64 µg/mL), meropenem (0.03–16 µg/mL), and ciprofloxacin (0.015–8 µg/mL). The quality control of the batch was performed with *E. coli* ATCC^®^ 25922. The bacterial inoculum was prepared as described for *Vibrio* spp. but with 0.9% NaCl solution. 

The epidemiological cut-off value (ECOFF) indicated in the Commission Implementing Decision (EU) 2020/1729 [18] was used as the interpretation threshold of AMR, except for colistin and tigecycline, for which the values defined for *Enterobacteriales* in the EUCAST clinical breakpoint table [31] were chosen, whereas the MIC breakpoints stated in CLSI document M100 [32] were used for azithromicyn and sulfamethoxazole (MIC values for sulphonamides). Multidrug resistance (MDR) was defined as nonsusceptibility to at least one agent in three or more antimicrobial categories [33]. 

Finally, the multiple antibiotic resistance (MAR) index was calculated as the ratio between the number of antibiotics to which a strain was resistant to and the total number of antibiotics used [34].

## 3. Results

### 3.1. Salmonella Isolates and Their Antimicrobial Resistance Profile

Among the 296 live bivalve molluscs analysed, only 1.7% of them were contaminated with *Salmonella* spp. Overall, five strains were isolated and tested for susceptibility to antimicrobial agents: three strains from clams (*S. enterica* subsp. *enterica* Kasenyi, *S. enterica* subsp. *enterica* Typhimurium, and *S. bongori* 48:z35:-) and two strains from mussels (*S. enterica* subsp. *enterica* Fischerhütte and *S. enterica* subsp. *enterica* Typhimurium). All isolates were sensitive to almost all antibiotics, except for S. Typhimurium isolated from *Venus gallina*, which showed resistance to sulfamethoxazole, trimethoprim, tetracycline, gentamicin, and ampicillin. Instead, S. Kasenyi and S. *bongori* 48:z35:- were resistant only to sulfamethoxazole.

### 3.2. Vibrio Isolates and Their Antimicrobial Resistance Profile

**Figure 2:**
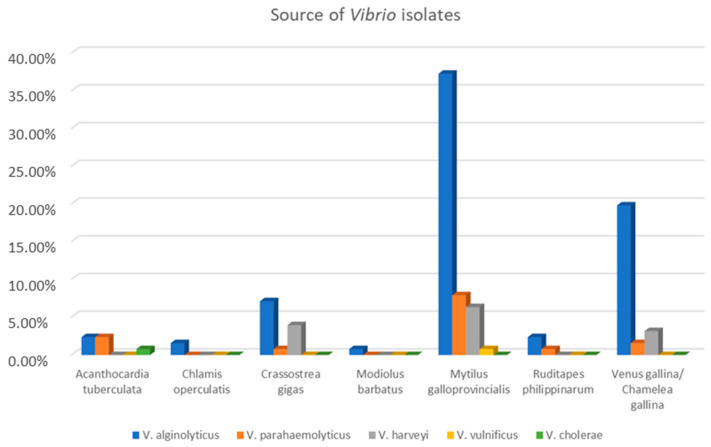
The percentages of *Vibrio* species isolated from each shellfish.

In total, 126 *Vibrio* strains (mostly *V. alginolyticus* and *V. parahaemolyticus*) were detected, with 38.2% (113/296) of shellfish samples contaminated with them. The API 20E system and the MALDI-TOF MS identification results were consistent, whereas the API 20NE displayed disputable and unreliable results. Furthermore, it is well known that the biochemical tests are inadequate for an accurate identification of *V. harveyi* [12,35] and that profiles for *V. harveyi* are not included in the bioMérieux database; thus, the API 20E and API 20NE systems do not allow this species to be properly identified via Apiweb™ and the identification of *V. harveyi* in this study was provided by the MALDI-TOF MS system. *Vibrio* spp. strains have been detected in shellfish collected from all sampling stations, with the exception of point 13. The temperature range for all isolates was of 9.4–29.9 °C, but isolation of potentially enteropathogenic species (*V. cholerae*, *V. parahaemolyticus*, and *V. vulnificus*) occurred when temperature ranged between 19.5 and 29.9 °C. The correlation between source and *Vibrio* species is shown in Figure 2. *V. alginolyticus* was the most prevalent species, found mostly in *Mytilus galloprovincialis*, followed by *Venus gallina/Chamelea gallina*.

No pathogenic genes were found in *V. cholerae* and *V. parahaemolyticus* isolates, except for one *V. parahaemolyticus* strain harbouring *trh* gene. The highest MAR index values for each sampling point are reported in Table 2 by specifying *Vibrio* species, resistance pattern, and source.

More specific details for each isolate about origin, resistance pattern, and MAR index are reported in Supplementary Table S1.

None of the 126 *Vibrio* isolates showed resistance to AZI, CHL, TET, AUG2, GEN, STR, AMI, or LEVO. Despite the interpretive criteria for *Vibrio* spp. other than *V. cholerae* provided in CLSI M45 guidelines [26] being uncertain for azithromycin, one *V. harveyi* (MIC = 4 µg/mL) could be surely stated as nonsusceptible and belonging to at least the intermediate category. Moreover, one *V. parahaemolyticus* displayed intermediate resistance to TET, one *V. parahaemolyticus* and five *V. alginolyticus* to AUG2, while intermediate resistance to STR was observed in all *Vibrio* species. The intermediate resistance percentages for each *Vibrio* species are detailed in Table 3. 

High resistance percentages to FIS (57.1%; 72/126) (MIC > 256 µg/mL), AMP (85.7%; 108/126), and FAZ (56.3%; 71/126) were found among all *Vibrio* species, except for *V. vulnificus* and *V. cholerae*, which were sensitive to AMP. Moreover, 38% and 24.6% of isolates were resistant (MIC > 64 µg/mL) and intermediate resistant (MIC = 32–64 µg/mL) to PIP, respectively. Resistance to FOX was expressed only by one *V. alginolyticus* isolate, although four *V. alginolyticus*, one *V. vulnificus*, one *V. cholerae*, and one *V. parahaemolyticus* were intermediate resistant. Resistance to MERO, SXT, PIP, P/T4, IMI, TAZ, A/S2, and FEP was observed only in *V. alginolyticus* strains with low resistance percentages, as results show in Table 4. 

Among *V. parahaemolyticus* strains, 94.1% (16/17) were resistant to FAZ, 76.5% (13/17) to AMP, 70.6% (12/17) to FIS, and 35.3% (6/17) to PIP. Susceptibility to all tested antimicrobials, except for FAZ, was found in the *V. parahaemolyticus trh*+ strain. 

Overall, 41.3% (52/126) of strains displayed MDR, mostly towards sulphonamides, penicillin, and cephems. The most prevalent MDR profile was FIS-AMP-PIP-FAZ, followed by FIS-AMP-FAZ, both of them found primarily in *V. alginolyticus* and secondarily in *V. parahaemolyticus.*

The MIC values are shown in Supplementary Table S2, including those relating to the antibiotic agents without available specific CLSI breakpoints.

## 4. Discussion

Overall, few shellfish samples (5/296) have been found to be contaminated with *Salmonella* spp. in the current study and only one S. Typhimurium strain showed multidrug resistance. Despite the low prevalence of *Salmonella*, the detection of pathogenic serovars raises concerns about consumer health, especially given the recent finding of S. Typhimurium strains of human origin, collected from the same region and resistant mainly to ampicillin, tetracycline, azithromycin, and sulfamethoxazole [17], which are the same antimicrobials depicted in our multidrug-resistant strain, except for azithromycin replaced with gentamicin. Giacometti et al. [36] carried out a similar investigation on 102 *Salmonella* isolates from bivalve molluscs and water samples collected during the official monitoring programme in the area of the province of Ferrara (northwestern area of the Adriatic Sea) between 2001 and 2017. The most common resistances observed by the authors were to streptomycin (58.8%), ampicillin (52%), and tetracycline (45.1%), whereas 44.12% of isolates were MDR. Thus, it seems to suggest that resistance to these antimicrobial classes (penicillin, aminoglycoside, and tetracycline) is widely spread in the Adriatic Sea, in accordance with the AMR profile exhibited by our S. Typhimurium strain. However, any comparison with our results is difficult due to the long period of sampling (7 years), the type of samples (molluscs and water), and the method used to evaluate the AMR (agar disk diffusion). It is easy to understand that the AMR patterns vary according to the geographical origin of the samples; indeed, results concerning 27 *Salmonella* strains isolated by Lozano-Leon et al. [37] from mussel samples harvested in Galicia are completely different: all isolates showed MDR and were resistant to cefuroxime and cefuroxime/axetil; the majority of them expressed resistance to cefoxitin and gentamicin and some resistance was observed towards ampicillin, amikacin, cephalothin, and tobramycin, although the antimicrobial susceptibility was performed only on some strains. 

As regards *Vibrio* spp., three different identification techniques were performed simultaneously in the present work to obtain a more reliable result, given that differentiation of closely related *Vibrio* species can be difficult. Although MALDI TOF MS is considered a valid tool, its discriminative power depends on the fullness of the reference library, which mainly contains clinically relevant species; thus, the reference library could be increased by inserting environmental bacterial isolates, especially marine bacteria, to make the MALDI TOF MS result more consistent. The API 20E and MALDI TOF MS systems gave the same results, except for the *V*. *harveyi* species because of the lack in the bioMérieux database. Therefore, the MALDI TOF identification of this *Vibrio* species was considered reliable and might perhaps be supported by biomolecular analysis, such as whole genome sequencing, which offers a great identification accuracy. *Vibrio* detection occurred during the entire investigation period but it is worth noting that potentially enteropathogenic species were isolated when water temperature was above 19.5 °C. It is well known that occurrence and densities of *V. parahaemolyticus* in molluscs are positively correlated to water temperatures [38] and outbreaks occur mainly during the warmer months in temperate zones [39]. However, the increase in surface temperature in coastal European seas in recent years has been linked to outbreaks caused by *Vibrio cholerae* nonO1-nonO139, *V. parahaemolyticus*, and *V. vulnificus* in several European countries [40]. Among potentially enteropathogenic species, *V. parahaemolyticus* was the most frequent one, isolated mainly from *Mytilus galloprovincialis*, but the only strain harbouring *trh* gene, hence proving to be harmful to humans, was isolated from *Venus gallina o Chamelea gallina*. *V. cholerae* isolation from *Acanthocardia tuberculata* cockles is a rare finding and certainly noteworthy given the scarcity of studies concerning this shellfish, mostly carried out in Morocco. For example, Boutaib et al. [41] found *Salmonella* spp. and non-pathogenic *Vibrio parahaemolyticus* in seven and five *Acanthocardia tuberculata* samples, respectively, but no results concerned *V. cholerae*, since this species was not included in the study.

In the current survey, the majority of *Vibrio* strains displayed resistance to ampicillin, sulfisoxazole, and cefazolin. Resistance to ampicillin was prevalent, as in previous studies [1,2,42,43]. 

If we compare our results with similar studies previously conducted in Italy, data seem to be quite varied. For instance, in the study carried out by Ottaviani et al. in 2001 [44] on several *Vibrio* species isolated from seafood, all strains exhibited susceptibility to imipenem, meropenem, chloramphenicol, and tetracycline, except 58% of *V. alginolyticus*, which were resistant to tetracycline; more than 90% of isolates showed susceptibility to oxolinic acid, cefotaxime, flumequine, doxycycline, and trimethoprim–sulphamethoxazole and more than 80% to nalidixic acid and ciprofloxacin. Moreover, resistance to streptomycin and lincomycin was found in more than 90% of isolates and many strains of *V*. *alginolyticus*, *V*. *harveyi*, *V*. *vulnificus*, and *V*. *parahaemolyticus* were resistant to penicillin, carbenicillin, ampicillin, cephalothin, and kanamycin, while *V*. *alginolyticus* and *V*. *parahaemolyticus* strains were resistant to rifampicin. A later investigation of antimicrobial resistance in *V. parahaemolyticus* from indigenous bivalves collected from harvesting areas along Italian coasts of the south Adriatic Sea, the central Tyrrhenian Sea, and the central Adriatic Sea reported that all isolates were resistant to ampicillin and amoxicillin but no resistances were observed to chloramphenicol, tetracycline, oxytetracycline, doxycycline, and trimethoprim/sulfamethoxazole [45]. Some strains exhibited resistance to cefotaxime (24.1%), cefalothin (43.7%), cefalexin (67.8%), colistin sulphate (13.8%), erythromycin (20.7%), and streptomycin (32.2%), whereas very low resistance percentages were found towards polymyxin B, nalidixic acid, oxolinic acid, nitrofurantoin, ciprofloxacin, kanamycin, and neomycin. Lopatek et al. [28] investigated the antimicrobial susceptibility of *V. parahaemolyticus* strains isolated from different species of raw shellfish and marine fish originated from various countries. They found that the majority of the strains were resistant to ampicillin and streptomycin and were recovered mainly from Italian samples; resistance to gentamicin was found in 12.5% of the strains, isolated from Italian, Dutch, and Norwegian samples, whereas only one strain was resistant to ciprofloxacin, isolated from Italian clams. 

It should be noted that the majority of the studies on AMR in *Vibrio* spp. performed the agar disk diffusion method; thus, few works can be properly compared with our results. 

In the current study, the *V. vulnificus* strain exhibited a poor resistance pattern, showing resistance only to cefazolin and an intermediate resistance to cefoxitin. However, Baker-Austin et al. [46] found numerous coastal and, also, septicaemia isolates in the USA resistant to antibiotics routinely prescribed for *V. vulnificus* infections, such as doxycycline, tetracycline, aminoglycosides, and cephalosporins, thus suggesting the importance of continued monitoring. In contrast, Bier et al. [27] reported that most antimicrobial agents recommended for treatment of *V. vulnificus* and *V. cholerae* non-O1/non-O139 infections were effective in vitro; likewise, in our study, both *V. vulnificus* and *V. cholerae* isolates did not display any worrisome resistance. 

Banerjee and Farber [42] characterised 1021 *Vibrio* strains isolated from molluscs harvested in Canada between 2006 and 2012 and found that only 4.9% of them were sensitive to all tested drugs, while the antibiotics contributing the most to AMR were ampicillin, cephalothin, erythromycin, kanamycin, and streptomycin, although a declining trend in the frequency of MDR/AMR *Vibrio* spp. was registered until 2012.

Recently, Chahouri et al. [47] conducted a similar microbiological investigation in the Agadir Bay (Morocco), performing the search and AMR characterisation of *Vibrio* and *Salmonella* strains from mussels, sediment, and water samples. In accordance with our results, they isolated *Vibrio* strains at a high frequency, while a low percentage was noted for *Salmonella*. The eight *Salmonella* isolates showed resistance to ampicillin (100%), chloramphenicol (87.5%), and amoxicillin/clavulanic acid (62.5%) but were sensitive to all the other antibiotics used. *Vibrio* strains were mainly resistant to ampicillin (57.7%), cephalothin (62%), amikacin (60.6%), and, to a lesser extent, to ciprofloxacin (26.8%). Moreover, the authors perfectly agree with us when they highlight the importance of environmental survey to properly assess the microbiological quality in aquatic ecosystems.

In our study, almost all *Vibrio* strains exhibited MAR index values ranging from 0.064 to 0.193, below the arbitrary value of 0.2, indicating low-risk contamination sites [34], with the only exception of one *V. alginolyticus* isolate, which was resistant to seven antibiotics (MAR = 0.225). Since indices between 0.20 and 0.25 are in a range of ambiguity [34] and no *Vibrio* strains displayed MAR indices above 0.25, it could be stated that isolates originated from a low-risk environment where antibiotics are not regularly used perhaps. Moreover, resistance was observed mostly towards unusual antimicrobial compounds, such as ampicillin, piperacillin, sulfisoxazole, and the first-generation cephalosporin cefazolin, while few strains exhibited resistance to trimethoprim/sulfamethoxazole, piperacillin/tazobactam, and ampicillin/sulbactam (five, two, and one *V. alginolyticus*, respectively), proving that the antimicrobial combination therapy is still quite effective. In conclusion, the level of AMR in the tested *Salmonella* and above all *Vibrio* strains, which represent a consistent bacterial population in the marine environment, seems to indicate a poor diffusion of this phenomenon in the investigated area, suggesting that the actual condition is not highly worrying but perhaps promising, especially in view of the strategies currently implemented for the prudent use of antimicrobials in both human medicine and the zootechnical sector. However, our findings are not negligible, as some *V. alginolyticus* isolates exhibited resistance towards the critically important antimicrobials for human medicine. The epidemiological value of our study is of great relevance given the methodical approach used for monitoring: the survey was conducted over a period of 19 months and in a rather large area particularly devoted to shellfish farming and shellfish harvesting. Thus, the large amount of systematic data collected give an overview of the current scenario in one of the most representative regions for the Italian shellfish production sector [48].

The present study, like other previous ones, suggests the opportunity to implement a national/European programme to monitor the prevalence and distribution of antimicrobial resistance pattern in several not only pathogenic, but also environmental bacteria. Special consideration should be given to bacteria isolated from seafood which are generally eaten raw or undercooked and could represent a great threat to human health by transferring mobile genetic elements for antibiotic resistance to human pathogens. With regard to the latter aspect, it is worth mentioning, for example, the isolation of a *V. parahaemolyticus* strain carrying the *bla*_NDM-1_ gene from seafood and displaying in vitro carbapenemase activity but not phenotypical resistance [49]; this is a certainly highly worrisome finding, which could lead to therapeutic failure; thus, further research should be encouraged. Investigation of AMR in microorganisms within a specific area is necessary to formulate effective antibiotic reduction programmes. Indeed, although antibiotic resistance is an ancient and natural phenomenon, it is generally recognised that it occurs mainly in bacteria inhabiting the gastrointestinal tract of subjects receiving antibiotics and that distribution of antibiotic resistance genes (ARGs) in the aquatic ecosystem reflects mostly the faecal contamination by ARB. Furthermore, antibiotic pollution contributes to promoting the emergence and maintenance of ARGs and ARB in a delimited area [50]. Hence, insightful information on trends of AMR distribution in site-specific microbial populations is fundamental to better understand the local use and abuse of antibiotics and realise appropriate corrective measures. In this perspective, it would be appropriate to fix MIC breakpoints, also, for environmental *Vibrio* species and our results could be useful to upgrade the MIC distribution for *Vibrio* spp. relating to the antibiotic agents without available specific CLSI breakpoints.

## Figures and Tables

**Table 1:** Number and type of samples for each sampling point.

Sampling Station	Area	Shellfish Species	*n*. Samples
1	Northern coast of Gargano	*Mytilus galloprovincialis*	12
2	Northern coast of Gargano	*Mytilus galloprovincialis*	13
3	Northern coast of Gargano	*Crassostrea gigas*	13
4	Northern coast of Gargano	*Mytilus galloprovincialis*	13
5	Northern coast of Gargano	*Mytilus galloprovincialis*	15
6	Varano lake	*Ruditapes philippinarum*	6
		*Mytilus galloprovincialis*	14
7	Varano lake	*Crassostrea gigas*	14
9	Varano lake	*Mytilus galloprovincialis*	13
10	Varano lake	*Mytilus galloprovincialis*	14
11	Southern coast of Gargano	*Crassostrea gigas*	6
		*Mytilus galloprovincialis*	11
12	Southern coast of Gargano	*Mytilus galloprovincialis*	14
13	Southern coast of Gargano	*Modiolus barbatus*	7
15	Southern coast of Gargano	*Acanthocardia tuberculata*	8
		*Modiolus barbatus*	1
16	Southern coast of Gargano	*Acanthocardia tuberculata*	8
17	Southern coast of Gargano	*Acanthocardia tuberculata*	8
18	Coastline of BAT ^1^ Province	*Venus gallina/Chamelea gallina*	17
19	Coastline of BAT ^1^ Province	*Venus gallina/Chamelea gallina*	15
20	Coastline of BAT ^1^ Province	*Venus gallina/Chamelea gallina*	17
21	Coastline of BAT ^1^ Province	*Venus gallina/Chamelea gallina*	12
22	Coastline of BAT ^1^ Province	*Venus gallina/Chamelea gallina*	12

^1^ BAT: Barletta-Andria-Trani.

**Table 2:** The highest values of multiple antibiotic resistance (MAR) index of *Vibrio* isolates.

Sampling Station	Matrix	Vibrio Species	Resistance Pattern	MAR Index
1	*Mytilus galloprovincialis*	*V. alginolyticus*	MERO, FIS, AMP, P/T4, PIP, FEP	0.193
2	*Mytilus galloprovincialis*	*V. alginolyticus*	FIS, SXT, AMP, PIP, FAZ	0.161
3	*Crassostrea gigas*	*V. alginolyticus*	FIS, AMP, P/T4, PIP, FAZ, TAZ, FEP	0.225
4	*Mytilus galloprovincialis*	*V. alginolyticus*	FIS, AMP, PIP, FAZ	0.129
		*V. harveyi*	FIS, AMP, PIP, FAZ	0.129
5	*Mytilus galloprovincialis*	*V. alginolyticus*	FIS, AMP, PIP, FAZ	0.129
		*V. alginolyticus*	FIS, AMP, PIP, FAZ	0.129
6	*Mytilus galloprovincialis*	*V. alginolyticus*	FIS, AMP, PIP, FAZ	0.129
		*V. parahaemolyticus*	FIS, AMP, PIP, FAZ	0.129
	*Ruditapes philippinarum*	*V. alginolyticus*	FIS, AMP, PIP, FAZ	0.129
		*V. alginolyticus*	FIS, AMP, PIP, FAZ	0.129
7	*Crassostrea gigas*	*V. harveyi*	AMP, PIP	0.064
9	*Mytilus galloprovincialis*	*V. parahaemolyticus*	FIS, AMP, PIP, FAZ	0.129
10	*Mytilus galloprovincialis*	*V. alginolyticus*	FIS, AMP, PIP, FAZ	0.129
		*V. alginolyticus*	FIS, AMP, PIP, FAZ	0.129
		*V. parahaemolyticus*	FIS, AMP, PIP, FAZ	0.129
11	*Mytilus galloprovincialis*	*V. vulnificus*	FIS, FAZ	0.064
12	*Mytilus galloprovincialis*	*V. alginolyticus*	FIS, AMP, FAZ	0.096
		*V. alginolyticus*	FIS, AMP, FAZ	0.096
		*V. alginolyticus*	FIS, AMP, FAZ	0.096
15	*Acanthocardia tuberculata*	*V. alginolyticus*	FIS, AMP, FAZ	0.096
16	*Acanthocardia tuberculata*	*V. parahaemolyticus*	FIS, AMP, FAZ	0.096
17	*Acanthocardia tuberculata*	*V. alginolyticus*	AMP, PIP, FAZ	0.096
18	*Venus gallina/ Chamelea gallina*	*V. alginolyticus*	FIS, AMP, PIP, FAZ	0.129
19	*Venus gallina/ Chamelea gallina*	*V. alginolyticus*	FIS, AMP, PIP, FAZ	0.129
20	*Venus gallina/ Chamelea gallina*	*V. alginolyticus*	FIS, AMP, PIP, FAZ	0.129
21	*Venus gallina/ Chamelea gallina*	*V. alginolyticus*	FIS, AMP, PIP, FAZ	0.129
22	*Venus gallina/ Chamelea gallina*	*V. harveyi*	FIS, AMP, PIP, FAZ	0.129
		*V. parahaemolyticus*	FIS, AMP, PIP, FAZ	0.129
		*V. alginolyticus*	FIS, AMP, PIP, FAZ	0.129

**Table 3:** Intermediate profile to single antimicrobial agents in percentage. The antimicrobial agents for which no intermediate resistance was found are not shown.

*Vibrio* Species	*n*	Antimicrobial Agents									
		FOX	AZI	TET	AUG2	STR	P/T4	PIP	FAZ	TAZ	A/S2
*V. alginolyticus*	90	4.4			5.6	3.3	1.1	26.7	30	1.1	6.7
*V. parahaemolyticus*	17	5.9		5.9	5.9	5.9		11.8			
*V. harveyi*	17		5.9			17.6		29.4	11.8		
*V. vulnificus*	1	100				100					
*V. cholerae*	1	100				100					

*n*: number of isolates.

**Table 4:** Resistance percentages to single antimicrobial agents. The antimicrobial agents for which no resistance was found are not shown.

Vibrio Species	*n*	Antimicrobial Agents											
		FOX	MERO	FIS	SXT	AMP	P/T4	IMI	PIP	FAZ	TAZ	A/S2	FEP
*V. alginolyticus*	90	1.1	1.1	61.1	5.6	93.3	2.2	1.1	42.2	55.6	1.1	1.1	3.3
*V. parahaemolyticus*	17			70.6		76.5			35.3	94.1			
*V. harveyi*	17			17.6		64.7			23.5	17.6			
*V. vulnificus*	1			100						100			
*V. cholerae*	1			100						100			

*n*: number of isolates.

## Data Availability

Not applicable.

## Supplementary Materials

The following supporting information can be downloaded at: https://www.mdpi.com/article/10.3390/microorganisms11020450/s1, Table S1. Information on Vibrio isolates: origin, resistance profile and MAR index. Table S2: Results for the broth microdilution assays of *Vibrio* isolates.

## References

[1] Shakerian A., Barton M.D., Akinbowale O.L., Khamesipour F. (2018). Antimicrobial resistance profile and resistance genes of Vibrio species isolated from giant freshwater prawn (*Macrobrachium Rosenbergii*) raised in Iran. J. Hellenic. Vet. Med. Soc..

[2] Sudha S., Mridula C., Silvester R., Hatha A.A.M. (2014). Prevalence and antibiotic resistance of pathogenic Vibrios in shellfishes from Cochin market. Indian J. Geo-Mar. Sci..

[3] Cabello F.C., Godfrey H.P., Tomova A., Ivanova L., Dölz H., Millanao A., Buschmann A.H. (2013). Antimicrobial use in aquaculture re-examined: Its relevance to antimicrobial resistance and to animal and human health. Environ. Microbiol..

[4] Baker-Austin C., Oliver J.D., Alam M., Ali A., Waldor M.K., Quadri F., Martinez-Urtaza J. (2018). *Vibrio* spp. infections. Nat. Rev. Dis. Primers.

[5] Rivera I.N., Chun J., Huq A., Sack R.B., Colwell R.R. (2001). Genotypes associated with virulence in environmental isolates of *Vibrio cholerae*. Appl. Environ. Microbiol..

[6] Dutta D., Kaushik A., Kumar D., Bag S. (2021). Foodborne pathogenic Vibrios: Antimicrobial resistance. Front. Microbiol..

[7] Mok J.S., Ryu A., Kwon J.Y., Kim B., Park K. (2019). Distribution of *Vibrio* species isolated from bivalves and bivalve culture environments along the Gyeongnam coast in Korea: Virulence and antimicrobial resistance of *Vibrio parahaemolyticus* isolates. Food Control.

[8] Ashrafudoulla M., Mizan M.F.R., Park H., Byun K.H., Lee N., Park S.H., Ha S.D. (2019). Genetic relationship, virulence factors, drug resistance profile and biofilm formation ability of *Vibrio parahaemolyticus* isolated from mussel. Front. Microbiol..

[9] Ina-Salwany M.Y., Al-Saari N., Mohamad A., Mursidi F.A., Mohd-Aris A., Amal M.n.A., Kasai H., Mino S., Sawabe T., Zamri-Saad M. (2019). Vibriosis in fish: A review on disease development and prevention. J. Aquat. Anim. Health.

[10] Labella A., Gennari M., Ghidini V., Trento I., Manfrin A., Borrego J.J., Lleo M.M. (2013). High incidence of antibiotic multi-resistant bacteria in coastal areas dedicated to fish farming. Mar. Pollut. Bull..

[11] Brehm T.T., Berneking L., Rohde H., Chistner M., Schlickewei C., Sena Martins M., Schmiedel S. (2020). Wound infection with *Vibrio harveyi* following a traumatic leg amputation after a motorboat propeller injury in Mallorca, Spain: A case report and review of literature. BMC Infect. Dis..

[12] Montánchez I., Kaberdin V.R. (2020). *Vibrio harveyi*: A brief survey of general characteristics and recent epidemiological traits associated with climate change. Mar. Environ. Res..

[13] (2003). Directive 2003/99/EC of the European Parliament and of the Council of 17 November 2003 on the Monitoring of Zoonoses and Zoonotic Agents, Amending Council Decision 90/424/EEC and Repealing Council Directive 92/117/EEC. http://data.europa.eu/eli/dir/2003/99/2013-07-01.

[14] European Food Safety Authority and European Centre for Disease Prevention and Control (2021). The European Union One Health 2019 Zoonoses Report. EFSA J..

[15] Maggi P., Carbonara S., Fico C., Santantonio T., Romanelli C., Sforza E., Pastore G. (1997). Epidemiological, clinical and therapeutic evaluation of the Italian cholera epidemic in 1994. Eur. J. Epidemiol..

[16] Rizzo G., Barbuti S., Leogrande G., Jatta E. (1975). Osservazioni sulla diagnosi batteriologica di colera e sulle caratteristiche degli stipiti isolati nella epidemia pugliese dell’estate 1973 [Studies on the bacteriological diagnosis of cholera and on the characteristics of isolated strains in the Apulia epidemic during the summer of 1973]. Ann. Sclavo.

[17] Alessiani A., Goffredo E., Mancini M., Occhiochiuso G., Faleo S., Didonna A., Fischetto R., Suglia F., De Vito D., Stallone A. (2022). Evaluation of antimicrobial resistance in *Salmonella* strains isolated from food, animal and human samples between 2017 and 2021 in southern Italy. Microorganisms.

[18] Commission Implementing Decision (EU) 2020/1729 of 17 November 2020 on the monitoring and reporting of antimicrobial resistance in zoonotic and commensal bacteria and repealing Implementing Decision 2013/652/EU, (2020). http://data.europa.eu/eli/dec_impl/2020/1729/oj.

[19] Regulation (EC) No 2160/2003 of the European Parliament and of the Council of 17 November 2003 on the control of salmonella and other specified food-borne zoonotic agents, (2003). http://data.europa.eu/eli/reg/2003/2160/2021-04-21.

[20] World Health Organization 2021 TrACSS Country Report on the Implementation of National Action Plan on Antimicrobial Resistance (AMR). https://cdn.who.int/media/docs/default-source/antimicrobial-resistance/amr-spc-npm/tracss/tracss-2021-italy.pdf?sfvrsn=10e5e354_4&download=true.

[21] Commission Implementing Regulation (EU) 2019/627 of 15 March 2019 laying down uniform practical arrangements for the performance of official controls on products of animal origin intended for human consumption in accordance with Regulation (EU) 2017/625 of the European Parliament and of the Council and amending Commission Regulation (EC) No 2074/2005 as regards official controls, (2019). http://data.europa.eu/eli/reg_impl/2019/627/2021-10-14.

[22] European Union Reference Laboratory for Monitoring of Marine Biotoxins (2021). Community Guide to the Principles of Good Practice for the Microbiological Classification and Monitoring of Bivalve Mollusc Production and Relaying Areas with Regard to Implementing Regulation 2019/627. https://www.aesan.gob.es/en/CRLMB/docs/docs/procedimientos/Micro_Control_Guide_DEC_2021.pdf.

[23] Commission Regulation (EC) No 2073/2005 of 15 November 2005 on microbiological criteria for foodstuffs, (2005). http://data.europa.eu/eli/reg/2005/2073/2020-03-08.

[24] (2017). Microbiology of the Food Chain—Horizontal Method for the Determination of Vibrio spp.—Part 1: Detection of Potentially Enteropathogenic Vibrio Parahaemolyticus, Vibrio Cholerae and Vibrio vulnificus.

[25] Mancini M.E., Beverelli M., Donatiello A., Didonna A., Dattoli L., Faleo S., Occhiochiuso G., Galante D., Rondinone V., Del Sambro L. (2022). Isolation and characterization of *Yersinia enterocolitica* from foods in Apulia and Basilicata regions (Italy) by conventional and modern methods. PLoS ONE.

[26] Clinical and Laboratory Standards Institute (CLSI) (2015). Methods for Antimicrobial Dilution and Disk Susceptibility Testing of Infrequently Isolated or Fastidious Bacteria.

[27] Bier N., Schwartz K., Guerra B., Strauch E. (2015). Survey on antimicrobial resistance patterns in *Vibrio vulnificus* and *Vibrio cholerae* non-O1/non-O139 in Germany reveals carbapenemase-producing *Vibrio cholerae* in coastal waters. Front. Microbiol..

[28] Lopatek M., Wieczorek K., Osek J. (2018). Antimicrobial resistance, virulence factors, and genetic profiles of *Vibrio parahaemolyticus* from seafood. Appl. Environ. Microbiol..

[29] (2020). Microbiology of the Food Chain—Horizontal Method for the Detection, Enumeration and Serotyping of Salmonella—Part 1: Detection of Salmonella spp.—Amendment 1: Broader Range of Incubation Temperatures, Amendment to the Status of Annex D, and Correction of the Composition of MSRV and SC.

[30] (2014). Microbiology of the Food Chain—Horizontal Method for the Detection, Enumeration and Serotyping of Salmonella—Part 3: Guidelines for Serotyping of Salmonella spp..

[31] European Committee on Antimicrobial Susceptibility Testing (EUCAST) Breakpoint Tables for Interpretation of MICs and Zone Diameters. Version 12.0..

[32] Clinical and Laboratory Standards Institute (CLSI) (2022). Performance Standards for Antimicrobial Susceptibility Testing. 32nd ed. CLSI Supplement M100.

[33] Magiorakos A.P., Srinivasan A., Carey R.B., Carmeli Y., Falagas M.E., Giske C.G., Harbarth S., Hindler J.F., Kahlmeter G., Olsson-Liljequist B. (2012). Multidrug-resistant, extensively drug-resistant and pandrug-resistant bacteria: An international expert proposal for interim standard definitions for acquired resistance. Clin. Microbiol. Infect..

[34] Krumperman P.H. (1983). Multiple antibiotic resistance indexing of *Escherichia coli* to identify high-risk sources of fecal contamination of foods. Appl. Environ. Microbiol..

[35] Stalin N., Srinivasan P. (2016). Molecular characterization of antibiotic resistant *Vibrio harveyi* isolated from shrimp aquaculture environment in the south east coast of India. Microb. Pathog..

[36] Giacometti F., Pezzi A., Galletti G., Tamba T., Merialdi G., Piva S., Serraino A., Rubini S. (2021). Antimicrobial resistance patterns in *Salmonella enterica* subsp. *enterica* and *Escherichia coli* isolated from bivalve molluscs and marine environment. Food Control.

[37] Lozano-León A., García-Omil C., Rodríguez-Souto R.R., Lamas A., Garrido-Maestu A. (2022). An evaluation of the pathogenic potential, and the antimicrobial resistance, of *Salmonella* strains isolated from mussels. Microorganisms.

[38] Shen X., Cai Y., Liu C., Liu W., Hui Y., Su Y.C. (2009). Effect of temperature on uptake and survival of *Vibrio parahaemolyticus* in oysters (*Crassostrea plicatula*). Int. J. Food Microbiol..

[39] Nair G.B., Ramamurthy T., Bhattacharya S.K., Dutta B., Takeda Y., Sack D.A. (2007). Global dissemination of *Vibrio parahaemolyticus* serotype O3:K6 and its serovariants. Clin. Microbiol. Rev..

[40] Le Roux F., Wegner K.M., Baker-Austin C., Vezzulli L., Osorio C.R., Amaro C., Ritchie J.M., Defoirdt T., Destoumieux-Garzón D., Blokesch M. (2015). The emergence of *Vibrio* pathogens in Europe: Ecology, evolution, and pathogenesis (Paris, 11–12th March 2015). Front. Microbiol..

[41] Boutaib R., Marhraoui M., Oulad Abdellah M.K., Bouchrif B. (2011). Comparative study on faecal contamination and occurrence of *Salmonella* spp. and *Vibrio parahaemolyticus* in two species of shellfish in Morocco. Open Environ. Sci..

[42] Banerjee S.K., Farber J.M. (2018). Trend and pattern of antimicrobial resistance in molluscan *Vibrio* species sourced to Canadian estuaries. Antimicrob. Agents Chemother..

[43] Håkonsholm F., Lunestad B.T., Aguirre Sánchez J.R., Martinez-Urtaza J., Marathe N.P., Svanevik C.S. (2020). Vibrios from the Norwegian marine environment: Characterization of associated antibiotic resistance and virulence genes. Microbiologyopen.

[44] Ottaviani D., Bacchiocchi I., Masini L., Leoni F., Carraturo A., Giammarioli M., Sbaraglia G. (2001). Antimicrobial susceptibility of potentially pathogenic halophilic vibrios isolated from seafood. Int. J. Antimicrob. Agents.

[45] Ottaviani D., Leoni F., Talevi G., Masini L., Santarelli S., Rocchegiani E., Susini F., Montagna C., Monno R., D’Annibale L. (2013). Extensive investigation of antimicrobial resistance *in Vibrio parahaemolyticus* from shellfish and clinical sources, Italy. Int. J. Antimicrob. Agents.

[46] Baker-Austin C., McArthur J.V., Lindell A.H., Wright M.S., Tuckfield R.C., Gooch J., Warner L., Oliver J., Stepanauskas R. (2009). Multi-site analysis reveals widespread antibiotic resistance in the marine pathogen *Vibrio vulnificus*. Microb. Ecol..

[47] Chahouri A., Radouane n., Yacoubi B., Moukrim A., Banaoui A. (2022). Microbiological assessment of marine and estuarine ecosystems using fecal indicator bacteria, *Salmonella*, *Vibrio* and antibiotic resistance pattern. Mar. Pollut. Bull..

[48] Istituto Superiore per la Protezione e la Ricerca Ambientale (ISPRA) Stato Dell’ambiente 84/2019. https://www.isprambiente.gov.it/public_files/annuario-2018.pdf.

[49] Briet A., Helsens N., Delannoy S., Debuiche S., Brisabois A., Midelet G., Granier S.A. (2018). NDM-1-producing *Vibrio parahaemolyticus* isolated from imported seafood. J. Antimicrob. Chemother..

[50] Haenni M., Dagot C., Chesneau O., Bibbal D., Labanowski J., Vialette M., Bouchard D., Martin-Laurent F., Calsat L., Nazaret S. (2022). Environmental contamination in a high-income country (France) by antibiotics, antibiotic-resistant bacteria, and antibiotic resistance genes: Status and possible causes. Environ. Int..

