# Supplementary material for: Systematic Survey of Vibrio spp. and Salmonella spp. in Bivalve Shellfish in Apulia Region (Italy): Prevalence and Antimicrobial Resistance
Source: Microorganisms. 2023 Feb 10;11(2):450. doi: 10.3390/microorganisms11020450 (PMC9966029; doi:10.3390/microorganisms11020450)
Supplement: Supplementary file 1 [file microorganisms-11-00450-s001.zip › microorganisms-2129918-supplementary.pdf]

Supplementary materials

Table S1. Information on Vibrio isolates: origin, resistance profile and MAR index.

Sampling station	Matrix	Isolate ID	Vibrio species	Resistance pattern	MAR index
1	*Mytilus galloprovincialis*	15518/2021-1	*V. alginolyticus*	FIS, AMP, FAZ	0.096
		15518/2021-2	*V. alginolyticus*	FIS	
		21506/2021-1	*V. alginolyticus*	MERO, FIS, AMP, P/T4, PIP, FEP	0.193
		22287/2021-1	*V. alginolyticus*	FIS, AMP, PIP, FAZ, A/S2	0.161
		20616/2022-1	*V. parahaemolyticus*	FIS, AMP, PIP, FAZ	0.129
2	*Mytilus galloprovincialis*	20656/2021-1	*V. alginolyticus*	FIS, SXT, AMP, PIP, FAZ	0.161
		21507/2021-1	*V. alginolyticus*	FIS, AMP, PIP	0.096
		22220/2021-1	*V. alginolyticus*	AMP, PIP, FAZ	0.096
		37738/2021-1	*V. harveyi*	–	
3	*Crassostrea gigas*	15030/2021-1	*V. alginolyticus*	FIS, AMP, FAZ	0.096
		20657/2021-1	*V. alginolyticus*	AMP, PIP	0.064
		20657/2021-2	*V. harveyi*	AMP	
		21508/2021-1	*V. alginolyticus*	AMP	
		22221/2021-1	*V. alginolyticus*	FIS, AMP, P/T4, PIP, FAZ, TAZ, FEP	0.225
		26755/2021-1	*V. harveyi*	AMP	
		37739/2021-1	*V. alginolyticus*	FIS, AMP, FAZ	0.096
		37739/2021-2	*V. harveyi*	–	
		16922/2022-2	*V. alginolyticus*	AMP	
4	*Mytilus galloprovincialis*	15517/2021-1	*V. alginolyticus*	AMP, FAZ	0.064
		22288/2021-1	*V. alginolyticus*	FIS, AMP, FAZ	0.096
		25806/2021-1	*V. alginolyticus*	AMP, FAZ	0.064
		27218/2021-1	*V. alginolyticus*	FIS, AMP, PIP, FAZ	0.129
		27218/2021-2	*V. harveyi*	FIS, AMP, PIP, FAZ	0.129
5	*Mytilus galloprovincialis*	15026/2021-1	*V. alginolyticus*	AMP	
		17571/2021-1	*V. alginolyticus*	FIS, AMP, PIP, FAZ	0.129
		17571/2021-2	*V. alginolyticus*	AMP, PIP, FAZ	0.096
		20658/2021-1	*V. parahaemolyticus*	AMP, PIP, FAZ	0.096
		21570/2021-1	*V. alginolyticus*	AMP, PIP, FAZ	0.096
		22222/2021-1	*V. alginolyticus*	FIS, AMP, PIP, FAZ	0.129
		22222/2021-2	*V. harveyi*	–	
		25803/2021-1	*V. alginolyticus*	–	
6	*Mytilus galloprovincialis*	3891/2021-1	*V. harveyi*	AMP	
		14651/2021-1	*V. harveyi*	AMP	
		21168/2021-1	*V. alginolyticus*	FIS, AMP, PIP, FAZ	0.129
		21249/2022-1	*V. parahaemolyticus*	FIS, AMP, FAZ	0.096
		24626/2022-1	*V. parahaemolyticus*	FIS, AMP, PIP, FAZ	0.129
	Ruditapes philippinarum	14662/2021-1	*V. alginolyticus*	FIS, AMP, FAZ	0.096
		17633/2021-1	*V. alginolyticus*	FIS, AMP, PIP, FAZ	0.129
		21169/2021-1	*V. alginolyticus*	FIS, AMP, PIP, FAZ	0.129
		21169/2021-2	*V. parahaemolyticus*	FIS, AMP, FAZ	0.096
7	*Crassostrea gigas*	14672/2021-1	*V. alginolyticus*	AMP	
		20312/2021-1	*V. harveyi*	–	
		21170/2021-1	*V. parahaemolyticus*	AMP	
		21170/2021-2	*V. alginolyticus*	FIS	
		26757/2021-1	*V. harveyi*	AMP, PIP	0.064
9		14683/2021-1	*V. alginolyticus*	FIS, AMP	0.064

	Mytilus galloprovincialis	17187/2022-1	*V. alginolyticus*	FIS, AMP	0.064
		19361/2022-1	*V. parahaemolyticus*	FAZ	
		21250/2022-1	*V. parahaemolyticus*	FAZ	
		24639/2022-1	*V. parahaemolyticus*	FIS, AMP, PIP, FAZ	0.129
		25214/2022-1	*V. parahaemolyticus*	FIS, AMP, FAZ	0.096
10	*Mytilus galloprovincialis*	14691/2021-2	*V. alginolyticus*	AMP	
		21171/2021-1	*V. alginolyticus*	FIS, AMP, PIP, FAZ	0.129
		17062/2022-1	*V. alginolyticus*	FIS, AMP, PIP, FAZ	0.129
		21252/2022-1	*V. parahaemolyticus*	FIS, AMP, FAZ	0.096
		25215/2022-1	*V. parahaemolyticus*	FIS, AMP, PIP, FAZ	0.129
11	*Crassostrea gigas*	26981/2021-1	*V. alginolyticus*	FEP	
		13873/2022-1	*V. harveyi*	AMP	
	Mytilus galloprovincialis	22158/2022-1	*V. vulnificus*	FIS, FAZ	0.064
12	*Mytilus galloprovincialis*	15516/2021-1	*V. alginolyticus*	FIS, AMP, FAZ	0.096
		19847/2021-1	*V. alginolyticus*	AMP, FAZ	0.064
		21259/2021-1	*V. alginolyticus*	FIS, AMP, FAZ	0.096
		24139/2021-1	*V. alginolyticus*	SXT, AMP	0.064
		24966/2021-1	*V. alginolyticus*	AMP, PIP	0.064
		12811/2022-1	*V. alginolyticus*	FIS, AMP, FAZ	0.096
		13874/2022-2	*V. alginolyticus*	FIS, AMP	0.064
15	*Acanthocardia tuberculata*	17488/2022-1	*V. alginolyticus*	FIS, AMP, FAZ	0.096
		22613/2022-1	*V. cholerae*	FIS, FAZ	0.064
		23090/2022-1	*V. parahaemolyticus*	FIS, FAZ	0.064
16	*Acanthocardia tuberculata*	17490/2022-1	*V. alginolyticus*	AMP, PIP	0.064
		22226/2022-1	*V. parahaemolyticus*	FIS, AMP, FAZ	0.096
		23093/2022-1	*V. parahaemolyticus*	FIS, FAZ	0.064
17	*Acanthocardia tuberculata*	17491/2022-1	*V. alginolyticus*	AMP, PIP, FAZ	0.096
18	*Venus gallina/ Chamelea gallina*	14076/2021-1	*V. alginolyticus*	FIS, AMP, FAZ	0.096
		17566/2021-1	*V. alginolyticus*	SXT, AMP, PIP	0.096
		21064/2021-1	*V. alginolyticus*	FIS, AMP	0.064
		21064/2021-2	*V. alginolyticus*	FIS, AMP	0.064
		17030/2022-1	*V. alginolyticus*	FIS, AMP, PIP, FAZ	0.129
19	*Venus gallina/ Chamelea gallina*	14077/2021-1	*V. alginolyticus*	AMP, FAZ	0.064
		21063/2021-1	*V. alginolyticus*	FIS, AMP, FAZ	0.096
		22996/2021-1	*V. harveyi*	AMP, PIP	0.064
		25306/2021-1	*V. alginolyticus*	FIS, AMP	0.064
		26424/2021-1	*V. alginolyticus*	AMP, FAZ	0.064
		27490/2021-1	*V. alginolyticus*	–	
		28792/2021-1	*V. alginolyticus*	AMP, PIP	0.064
		33798/2021-1	*V. harveyi*	–	
		10739/2022-1	*V. alginolyticus*	FIS, AMP	0.064
		17038/2022-1	*V. alginolyticus*	FIS, AMP, PIP, FAZ	0.129
20	*Venus gallina/ Chamelea gallina*	14078/2021-1	*V. alginolyticus*	AMP, FAZ	0.064
		19483/2021-1	*V. alginolyticus*	FIS, AMP	0.064
		25309/2021-1	*V. alginolyticus*	FIS, AMP, PIP, FAZ	0.129
		26425/2021-1	*V. alginolyticus*	AMP	
		10742/2022-1	*V. alginolyticus*	FIS, AMP, FAZ	0.096
21	*Venus gallina/ Chamelea gallina*	16579/2021-1	*V. alginolyticus*	FIS, SXT, AMP	0.096
		18687/2021-1	*V. alginolyticus*	FIS, AMP, PIP	0.096

		22986/2021-1	*V. parahaemolyticus*	AMP, FAZ	0.064
		22986/2021-2	*V. alginolyticus*	FIS, AMP, PIP, FAZ	0.129
22	*Venus gallina/ Chamelea gallina*	16588/2021-1	*V. harveyi*	FIS, AMP, PIP, FAZ	0.129
		18696/2021-1	*V. parahaemolyticus*	FIS, AMP, PIP, FAZ	0.129
		21611/2021-1	*V. alginolyticus*	AMP, PIP, FAZ	0.096
		22989/2021-1	*V. alginolyticus*	AMP, FAZ	0.064
		27065/2021-1	*V. alginolyticus*	FIS, AMP, PIP, FAZ	0.129
Various origins	*Chlamis operculatis*	16677/2021-1	*V. alginolyticus*	AMP	
		16677/2021-2	*V. alginolyticus*	AMP	
	Modiolus barbatus	19490/2021-1	*V. alginolyticus*	FIS, AMP, PIP, FAZ	0.129
	Mytilus galloprovincialis	9578/2021-1	*V. alginolyticus*	FIS, AMP, FAZ	0.096
		13421/2021-1	*V. harveyi*	FIS, AMP, FAZ	0.096
		13652/2021-1	*V. alginolyticus*	AMP	
		14537/2021-1	*V. harveyi*	AMP	
		16199/2021-1	*V. alginolyticus*	FIS, AMP	0.064
		16199/2021-2	*V. alginolyticus*	AMP	
		16365/2021-2	*V. alginolyticus*	AMP	
		16827/2021-1	*V. alginolyticus*	FIS, AMP, FAZ	0.096
		16827/2021-2	*V. alginolyticus*	FIS, AMP	0.064
		17417/2021-1	*V. alginolyticus*	FIS	
		18643/2021-1	*V. alginolyticus*	FIS, SXT, AMP, PIP, FAZ	0.161
		18821/2021-1	*V. alginolyticus*	FIS, AMP, PIP, FAZ	0.129
		19487/2021-1	*V. alginolyticus*	FIS, AMP, PIP, FAZ	0.129
		19852/2021-1	*V. alginolyticus*	FIS, AMP, PIP, FAZ	0.129
		19945/2021-1	*V. alginolyticus*	FIS, AMP, PIP, FAZ	0.129
		20008/2021-1	*V. alginolyticus*	FIS, AMP, PIP	0.096
		21015/2021-1	*V. alginolyticus*	FIS, AMP, FAZ	0.096
		21957/2021-1	*V. alginolyticus*	FIS, AMP, PIP	0.096
		22015/2021-1	*V. alginolyticus*	AMP, PIP, FAZ	0.096
		25101/2021-1	*V. harveyi*	–	
	Venus gallina/ Chamelea gallina	21062/2021-1	*V. alginolyticus*	AMP	

Various origin: samples collected from purification and dispatch centres or at retail as part of official control. MERO: meropenem; FIS: sulfisoxazole; SXT: trimethoprim/sulfamethoxazole; AMP: ampicillin; P/T4: piperacillin/tazobactam constant 4; PIP: piperacillin; FAZ: cefazolin; A/S2: ampicillin/sulbactam; TAZ: ceftazidime; FEP cefepime.

Table S2. Results for the broth microdilution assays of Vibrio isolates.

ID CEPPO	Specie	FOX	AZI	CHL	TET	AXO	AUG2	CIP	GEN	NAL	MERO	FIS	SXT	AMP	STR	AMI	P/T4	TGC	TIM2	LEVO	NIT	DOR	MIN	ETP	IMI	PIP	FAZ	TOB	TAZ	A/S2	AZT	FEP
3891/2021-1	*V. harveyi*	2	–	–	–	–	2/1	0,25	2	2	–	–	–	32	16	–	–	–	–	-	–	-	-	-	-	64	-	-	-	-	2	-
9578/2021-1	*V. alginolyticus*	4	–	–	–	–	4/2	–	1	–	–	>256	–	32	16	–	–	–	–	–	–	–	-	-	8	-	16	-	-	-	-	-
13421/2021-1	*V. harveyi*	4	4	–	–	–	–	0,12	1	–	–	>256	–	32	16	–	–	–	–	–	–	–	–	–	-	64	16	-	-	-	8	-
13652/2021-1	*V. alginolyticus*	2	–	–	–	–	–	–	0,5	16	–	64	–	32	4	–	–	–	–	–	–	–	-	-	-	-	4	-	-	-	-	-
14076/2021-1	*V. alginolyticus*	8	1	–	–	–	8/4	0,5	1	1	–	>256	0,25/4,75	32	16	–	–	–	64/2	–	–	–	-	-	-	64	16	-	-	8/4	2	-
14077/2021-1	*V. alginolyticus*	4	2	–	–	–	–	0,25	2	1	–	128	–	32	8	–	–	–	64/2	–	–	–	-	-	-	64	16	-	-	8/4	16	-
14078/2021-1	*V. alginolyticus*	4	–	–	–	–	4/2	0,12	1	1	–	64	–	32	8	–	–	–	–	–	–	–	-	-	-	-	8	-	-	-	2	-
14537/2021-1	*V. harveyi*	2	–	–	–	–	–	–	1	1	–	–	–	32	8	–	–	–	–	–	–	–	-	-	-	64	-	-	-	-	4	-
14651/2021-1	*V. harveyi*	1	0,5	–	–	–	–	0,12	1	-	–	–	–	32	8	–	–	–	–	–	–	–	-	-	-	64	2	-	-	-	2	-
14662/2021-1	*V. alginolyticus*	4	–	–	–	–	2/1	0,06	0,5	1	–	>256	0,25/4,75	32	8	–	–	–	–	–	–	–	-	-	-	64	8	-	-	-	-	-
14672/2021-1	*V. alginolyticus*	8	–	–	–	–	2/1	0,12	1	-	–	–	–	32	32	–	–	–	–	–	–	–	-	-	-	64	4	-	-	-	-	-

14683/2021-1	*V. alginolyticus*	4	–	–	–	–	2/1	0,06	1	1	–	>256	–	32	16	–	–	–	–	–	–	–	-	-	-	64	4	-	-	-	2	-
14691/2021-2	*V. alginolyticus*	8	0,5	–	–	–	4/2	–	1	-	–	–	–	32	8	–	–	–	–	–	–	–	-	-	-	-	-	-	-	8/4	2	-
15026/2021-1	*V. alginolyticus*	4	–	–	–	–	2/1	0,12	1	-	–	–	–	32	8	–	–	–	–	–	–	–	-	-	-	64	4	-	-	-	-	-
15030/2021-1	*V. alginolyticus*	4	–	–	–	–	2/1	0,12	0,5	1	–	>256	–	32	8	–	–	–	–	–	–	–	-	-	-	64	8	-	-	-	-	-
15516/2021-1	*V. alginolyticus*	2	–	–	–	–	–	–	1	1	–	>256	–	32	16	–	–	–	64/2	–	–	–	-	-	-	64	8	-	-	-	2	-
15517/2021-1	*V. alginolyticus*	4	0,5	–	–	0,5	8/4	0,5	1	4	–	64	–	32	8	–	–	–	64/2	–	–	–	-	-	-	64	16	-	-	8/4	2	-
15518/2021-1	*V. alginolyticus*	2	–	–	–	–	4/2	0,12	1	1	–	>256	–	32	16	–	–	–	32/2	–	–	–	-	-	-	64	8	-	-	-	-	-
15518/2021-2	*V. alginolyticus*	4	–	–	–	–	2/1	0,12	1	2	–	>256	–	–	16	–	–	–	–	–	–	–	-	-	-	-	4	-	-	-	-	-
16199/2021-1	*V. alginolyticus*	2	–	–	–	–	8/4	0,25	2	4	–	>256	–	32	8	–	–	–	64/2	-	-	-	-	-	-	64	4	-	-	8/4	2	-
16199/2021-2	*V. alginolyticus*	2	–	–	–	–	2/1	0,12	0,5	2	–	–	–	32	8	–	–	–	–	–	–	–	-	-	-	64	4	-	-	-	-	-
16365/2021-2	*V. alginolyticus*	2	–	–	–	–	–	0,12	0,5	2	–	64	–	32	8	–	–	–	–	–	–	–	-	-	-	64	4	-	-	-	16	-
16579/2021-1	*V. alginolyticus*	2	–	–	–	–	4/2	0,12	0,5	1	–	>256	4/76	32	8	–	–	–	–	–	–	–	-	-	-	64	4	-	-	-	2	-
16588/2021-1	*V. harveyi*	8	1	–	–	–	4/2	0,25	2	2	–	>256	0,25/4,75	32	32	–	–	–	32/2	–	–	–	-	-	-	>64	16	-	-	8/4	4	-
16677/2021-1	*V. alginolyticus*	1	–	–	–	–	–	0,06	0,5	2	–	32	–	32	8	–	–	–	–	–	–	–	-	-	-	-	-	-	-	-	-	-

16677/2021-2	*V. alginolyticus*	2	–	–	–	–	4/2	0,25	1	2	–	128	–	32	8	–	–	–	–	–	–	–	-	-	-	64	4	-	-	-	-	-
16827/2021-1	*V. alginolyticus*	4	–	–	–	–	4/2	0,12	–	2	–	>256	–	32	4	–	–	–	–	–	–	–	-	-	-	64	8	-	-	-	-	-
16827/2021-2	*V. alginolyticus*	2	–	–	–	–	–	0,25	0,5	2	–	>256	–	32	16	–	–	–	–	–	–	–	-	-	-	64	4	-	-	8/4	2	-
17417/2021-1	*V. alginolyticus*	2	–	–	–	–	–	0,06	–	2	–	>256	–	8	8	–	–	–	–	–	–	–	-	-	-	-	4	-	-	-	-	-
17566/2021-1	*V. alginolyticus*	1	–	–	–	–	8/4	0,25	1	2	–	64	4/76	32	16	–	–	–	16/2	–	–	–	-	-	-	>64	4	-	-	-	2	-
17571/2021-1	*V. alginolyticus*	4	–	–	–	–	4/2	-	0,5	-	–	>256	–	32	8	–	–	–	–	–	–	–	-	-	-	>64	8	-	-	-	4	-
17571/2021-2	*V. alginolyticus*	2	–	–	–	–	4/2	0,06	0,5	1	–	64	–	32	8	–	–	–	–	–	–	–	-	-	-	>64	8	-	-	-	-	-
17633/2021-1	*V. alginolyticus*	8	–	–	–	–	8/4	-	1	-	–	>256	–	32	8	–	–	–	64/2	–	–	–	-	-	-	>64	8	-	-	8/4	2	-
18643/2021-1	*V. alginolyticus*	4	–	–	–	–	8/4	-	1	1	–	>256	4/76	32	8	–	–	–	–	–	–	–	-	-	-	>64	8	> 8	-	-	-	-
18687/2021-1	*V. alginolyticus*	4	–	–	4	–	4/2	0,12	0,5	2	–	>256	–	32	8	–	–	–	–	–	–	–	-	-	-	>64	4	-	-	-	-	-
18696/2021-1	*V. parahaemolyticus*	8	–	–	–	–	–	-	1	-	–	>256	–	32	16	–	–	–	–	–	–	–	-	-	-	>64	8	-	-	-	2	-
18821/2021-1	*V. alginolyticus*	4	–	–	–	–	8/4	0,25	2	-	–	>256	–	32	8	–	–	–	>64/2	–	–	–	-	-	-	>64	8	-	-	16/8	2	-
19483/2021-1	*V. alginolyticus*	4	–	–	–	–	4/2	0,25	1	2	–	>256	–	32	8	–	–	–	–	–	–	–	-	-	-	32	4	-	-	-	-	-
19487/2021-1	*V. alginolyticus*	8	–	–	–	–	8/4	0,12	0,5	-	–	>256	–	32	4	–	–	8	–	–	–	–	-	-	-	>64	16	-	8	-	2	-

19490/2021-1	*V. alginolyticus*	4	–	–	–	–	8/4	0,12	0,5	2	–	>256	–	32	8	–	–	–	–	–	–	–	-	-	-	>64	8	-	-	-	2	-
19847/2021-1	*V. alginolyticus*	4	–	–	–	–	–	-	–	-	–	64	–	32	-	–	–	–	–	–	–	–	-	-	-	-	8	-	-	-	-	-
19852/2021-1	*V. alginolyticus*	8	–	–	–	–	8/4	0,25	2	1	–	>256	–	32	8	–	–	–	>64/2	–	–	–	-	-	-	>64	16	-	-	-	2	-
19945/2021-1	*V. alginolyticus*	2	–	–	–	–	2/1	0,12	–	2	–	>256	–	32	8	–	–	–	–	–	–	–	-	-	-	>64	8	-	-	-	-	-
20008/2021-1	*V. alginolyticus*	4	–	–	–	–	–	0,12	0,5	-	–	>256	–	32	8	–	–	–	–	–	–	–	-	-	-	>64	4	-	-	-	-	-
20312/2021-1	*V. harveyi*	-	–	–	–	–	–	–	–	–	–	–	–	–	–	–	–	–	–	–	–	–	-	-	-	-	-	-	-	-	-	-
20656/2021-1	*V. alginolyticus*	-	–	–	–	–	4/2	–	–	–	–	>256	4/76	32	4	–	–	–	–	–	–	–	-	-	-	>64	8	-	-	-	-	-
20657/2021-1	*V. alginolyticus*	4	–	–	–	–	–	0,06	0,5	–	–	–	–	32	8	–	–	–	–	–	–	–	-	-	-	>64	-	-	-	-	-	-
20657/2021-2	*V. harveyi*	2	–	–	–	–	–	–	1	–	–	128	–	32	16	–	–	–	–	–	–	–	-	-	-	-	2	-	-	-	2	-
20658/2021-1	*V. parahaemolyticus*	16	–	–	–	–	16/8	0,25	2	2	–	256	–	32	8	–	–	–	>64/2	–	–	–	-	-	-	>64	8	-	-	8/4	2	-
21015/2021-1	*V. alginolyticus*	8	–	–	–	–	2/1	0,06	1	1	–	>256	–	32	-	–	–	–	–	–	–	–	-	-	-	-	16	-	2	-	2	-
21062/2021-1	*V. alginolyticus*	2	–	–	–	–	–	0,12	0,5	2	–	64	–	32	8	–	–	–	–	–	–	–	-	-	-	-	2	-	-	16/8	-	-
21063/2021-1	*V. alginolyticus*	8	–	–	–	–	8/4	0,25	4	1	–	>256	–	32	32	–	–	–	64/2	–	–	–	–	-	-	64	8	-	-	-	2	-
21064/2021-1	*V. alginolyticus*	8	–	–	–	–	16/8	0,5	2	1	–	>256	–	32	8	–	–	–	64/2	–	–	–	–	-	-	64	-	-	-	16/8	16	-

21064/2021-2	*V. alginolyticus*	4	–	–	–	–	4/2	0,12	1	1	–	>256	–	32	16	–	–	–	–	–	–	–	–	-	-	-	-	-	-	-	-	-
21168/2021-1	*V. alginolyticus*	8	–	–	–	–	8/4	0,25	1	1	–	>256	–	32	8	–	–	–	>64/2	–	–	–	–	-	-	>64	16	-	-	-	2	-
21169/2021-1	*V. alginolyticus*	16	–	–	–	–	8/4	–	1	–	–	>256	–	32	8	–	–	–	32/2	–	–	–	–	-	-	>64	16	-	-	-	2	-
21169/2021-2	*V. parahaemolyticus*	8	–	–	–	–	4/2	0,06	–	–	–	>256	0,25/4,75	32	16	–	–	–	–	–	–	–	–	-	-	64	16	-	-	-	-	-
21170/2021-1	*V. parahaemolyticus*	2	–	–	–	–	–	0,12	2	1	–	64	–	32	16	–	–	–	–	–	–	–	–	-	-	-	-	-	-	-	-	-
21170/2021-2	*V. alginolyticus*	1	–	–	–	–	–	0,03	–	1	–	>256	–	2	8	–	–	–	–	–	–	–	–	-	-	-	-	-	-	-	-	-
21171/2021-1	*V. alginolyticus*	16	–	–	–	–	8/4	0,06	1	-	–	>256	0,5/9,5	32	8	–	–	–	>64/2	–	–	–	–	-	-	>64	16	-	-	16/8	2	-
21259/2021-1	*V. alginolyticus*	8	–	–	–	–	–	0,12	0,5	2	–	>256	–	32	8	–	–	–	–	–	–	–	–	-	-	-	8	-	-	-	16	-
21506/2021-1	*V. alginolyticus*	1	–	–	–	–	–	0,12	1	1	4	>256	–	32	8	–	128/4	-	64/2	–	–	–	–	1	2	>64	4	-	-	-	-	16
21507/2021-1	*V. alginolyticus*	4	–	–	–	–	8/4	0,03	–	–	–	>256	–	32	8	–	–	–	>64/2	–	–	–	–	-	-	>64	-	-	-	8/4	-	-
21508/2021-1	*V. alginolyticus*	2	–	–	–	–	–	0,06	–	–	–	64	–	32	8	–	–	–	–	–	–	–	–	-	-	-	4	-	-	-	-	-
21570/2021-1	*V. alginolyticus*	8	–	–	–	–	4/2	0,12	1	–	–	32	–	32	8	–	–	–	–	–	–	–	–	-	-	>64	16	-	-	-	2	-
21611/2021-1	*V. alginolyticus*	8	–	–	–	–	8/4	0,12	1	8	–	32	–	32	8	–	–	–	–	–	–	–	–	-	-	>64	8	-	-	-	4	-
21957/2021-1	*V. alginolyticus*	4	–	–	–	–	8/4	0,03	1	–	–	>256	–	32	8	–	–	–	–	–	–	–	–	-	-	>64	4	-	-	-	-	-

22015/2021-1	*V. alginolyticus*	8	–	–	–	–	16/8	0,06	0,5	1	–	–	–	32	4	–	–	–	–	–	–	–	–	-	-	>64	16	-	-	8/4	2	-
22220/2021-1	*V. alginolyticus*	4	–	–	–	–	8/4	0,12	2	–	–	32	–	32	8	–	–	–	>64/2	–	–	–	–	-	-	>64	16	-	-	16/8	2	-
22221/2021-1	*V. alginolyticus*	8	–	–	–	2	8/4	0,12	1	1	–	>256	1/19	32	8	–	>128/4	–	–	–	–	–	–	-	-	>64	16	-	16	-	8	16
22222/2021-1	*V. alginolyticus*	32	–	–	–	–	16/8	0,25	2	–	–	>256	0,5/9,5	32	16	–	–	–	>64/2	–	–	–	–	-	-	>64	>16	-	-	-	-	-
22222/2021-2	*V. harveyi*	-	–	–	–	–	–	–	–	–	–	–	–	16	-	–	–	–	–	–	–	–	–	-	-	-	-	-	-	-	-	-
22287/2021-1	*V. alginolyticus*	16	–	–	–	–	16/8	0,06	1	–	–	>256	–	32	8	–	–	–	>64/2	–	–	–	–	-	-	>64	>16	-	-	>16/8	2	-
22288/2021-1	*V. alginolyticus*	8	–	–	–	–	4/2	0,12	0,5	2	–	>256	–	32	8	–	–	–	–	–	–	–	–	-	-	-	16	-	-	-	2	-
22986/2021-1	*V. parahaemolyticus*	4	–	–	–	–	2/1	0,06	1	1	–	256	–	32	8	–	–	–	–	–	–	–	–	-	-	-	16	-	-	-	-	-
22986/2021-2	*V. alginolyticus*	4	–	–	–	–	4/2	0,06	–	1	–	>256	–	32	4	–	–	–	–	–	–	–	–	-	-	>64	8	-	-	-	16	-
22989/2021-1	*V. alginolyticus*	8	–	–	–	–	4/2	–	0,5	-	–	256	–	32	8	–	–	–	–	–	–	–	–	-	-	-	16	-	-	-	4	-
22996/2021-1	*V. harveyi*	4	–	–	–	–	2/1	0,25	2	-	–	–	–	32	32	–	–	–	16/2	–	–	–	–	-	-	>64	4	-	-	-	4	-
24139/2021-1	*V. alginolyticus*	4	–	–	–	–	2/1	0,25	0,5	2	–	256	>4/76	32	8	–	–	–	–	–	–	–	–	-	-	64	4	-	-	-	2	-
24966/2021-1	*V. alginolyticus*	2	–	–	–	–	2/1	0,12	0,5	1	–	–	–	32	8	–	–	–	–	–	–	–	–	0,5	-	>64	2	-	-	-	-	-
25101/2021-1	*V. harveyi*	-	–	–	–	–	–	–	–	–	–	–	–	–	-	–	–	–	–	–	–	–	–	-	-	-	-	-	-	-	-	-

25306/2021-1	*V. alginolyticus*	2	–	–	–	–	4/2	0,03	–	–	–	>256	–	32	-	–	–	–	–	–	–	–	–	-	-	64	-	-	-	-	-	-
25309/2021-1	*V. alginolyticus*	8	–	–	–	–	8/4	–	1	–	–	>256	–	32	8	–	–	–	>64/2	–	–	–	–	-	-	>64	16	-	-	16/8	-	-
25803/2021-1	*V. alginolyticus*	-	–	–	–	–	–	0,12	0,5	1	–	256	–	–	8	–	–	–	–	–	–	–	–	-	-	-	-	-	-	-	-	-
25806/2021-1	*V. alginolyticus*	8	–	–	–	–	4/2	0,06	0,5	1	–	32	–	32	16	–	–	–	–	–	–	–	–	-	-	-	16	-	-	-	2	-
26424/2021-1	*V. alginolyticus*	8	–	–	–	–	4/2	–	0,5	-	–	256	–	32	4	–	–	–	–	–	–	–	–	-	-	-	16	-	-	-	2	-
26425/2021-1	*V. alginolyticus*	4	–	–	–	–	–	0,12	0,5	1	–	64	–	32	8	–	–	–	–	–	–	–	–	-	-	-	4	-	-	-	-	-
26755/2021-1	*V. harveyi*	2	–	–	–	–	–	0,12	2	–	–	64	–	32	8	–	–	–	–	–	–	–	–	-	-	-	-	-	-	-	2	
26757/2021-1	*V. harveyi*	2	–	–	–	–	–	0,12	1	1	–	32	–	32	8	–	–	–	–	–	–	–	–	-	-	>64	-	-	-	-	-	-
26981/2021-1	*V. alginolyticus*	-	–	–	–	–	–	–	–	16	–	-	–	–	–	–	64/4	-	–	–	–	–	–	-	-	64		-	-	-	4	32
27065/2021-1	*V. alginolyticus*	8	–	–	–	–	16/8	0,12	2	2	–	>256	–	32	16	–	–	–	>64/2	–	–	–	–	-	-	>64	16	-	-	8/4	-	-
27218/2021-1	*V. alginolyticus*	16	–	–	–	–	4/2	0,06	1	–	–	>256	–	32	8	–	–	–	–	–	–	–	-	-	-	>64	>16	-	-	8/4	4	-
27218/2021-2	*V. harveyi*	4	1	–	–	–	2/1	0,25	2	1	–	>256	0,25/4,75	32	16	–	–	–	32/2	–	–	–	-	-	-	>64	8	-	-	-	8	-
27490/2021-1	*V. alginolyticus*	-	–	–	–	–	–	–	–	–	–	–	–	–	–	–	–	–	–	–	–	–	–	0,5	-	-	-	-	-	-	-	-
28792/2021-1	*V. alginolyticus*	4	–	–	8	–	0,12	0,5	1	–	–	–	–	32	8	–	–	–	-	–	–	–	-	-	-	>64	4	-	-	-	-	-

33798/2021-1	*V. harveyi*	4	–	–	–	–	-	0,25	1	–	–	–	–	4	8	–	–	–	-	–	–	–	-	-	-	-	-	-	-	-	4	-
37738/2021-1	*V. harveyi*	-	–	–	–	–	–	–	–	–	–	–	–	–	–	–	–	–	-	–	–	–	-	-	-	-	-	-	-	-	-	-
37739/2021-1	*V. alginolyticus*	8	–	–	–	–	4/2	–	2	–	–	>256	–	32	16	–	–	–	-	–	–	–	-	-	-	-	8	-	-	-	-	-
37739/2021-2	*V. harveyi*	-	–	–	–	–	-	–	-	–	–	–	–	–	–	–	–	–	–	–	–	–	-	-	-	-	-	4	-	-	-	-
10739/2022-1	*V. alginolyticus*	4	–	–	–	–	2/1	0,12	0,5	1	–	>256	–	32	8	–	–	–	-	–	–	–	-	-	-	-	4	-	-	-	2	-
10742/2022-1	*V. alginolyticus*	8	–	–	8	–	2/1	0,25	1	1	–	>256	0,25/4,75	32	8	–	–	–	-	–	–	–	-	-	-	32	8	-	-	-	2	-
12811/2022-1	*V. alginolyticus*	8	–	–	-	–	2/1	0,12	1	1	–	>256	–	32	8	–	–	–	>64/2	-	>64	-	2	-	-	-	8	-	-	-	4	-
13873/2022-1	*V. harveyi*	8	0,5	–	–	–	2/1	0,25	2	1	–	256	–	32	32	–	–	–	–	–	–	–	-	-	-	32	4	-	-	-	2	-
13874/2022-2	*V. alginolyticus*	4	–	–	–	–	2/1	0,12	1	1	–	>256	–	32	8	–	–	–	–	–	–	–	-	-	-	-	4	-	-	-	2	-
16922/2022-2	*V. alginolyticus*	4	–	–	–	–	4/2	0,25	0,5	1	–	64	–	32	8	–	–	–	–	–	–	–	-	-	-	-	4	-	-	-	-	-
17030/2022-1	*V. alginolyticus*	8	–	–	–	–	4/2	0,12	2	1	–	>256	0,25/4,75	32	32	–	–	–	–	–	–	–	-	-	-	>64	8	-	-	-	-	-
17038/2022-1	*V. alginolyticus*	8	0,5	–	–	–	4/2	0,25	1	1	–	>256	–	32	8	–	–	–	–	–	–	–	-	-	-	>64	8	-	-	-	2	-
17062/2022-1	*V. alginolyticus*	8	0,5	–	–	–	4/2	0,12	-	2	–	>256	0,25/4,75	32	8	–	–	–	–	–	–	–	-	-	-	>64	8	-	-	-	2	-
17187/2022-1	*V. alginolyticus*	4	0,5	–	–	–	4/2	0,12	0,5	1	–	>256	0,25/4,75	32	8	–	–	–	–	–	–	–	-	-	-	-	4	-	-	-	-	-

17488/2022-1	*V. alginolyticus*	8	–	–	–	–	8/4	0,12	0,5	1	–	>256	–	32	16	–	–	–	–	–	–	–	-	-	-	-	8	-	-	-	2	-
17490/2022-1	*V. alginolyticus*	8	–	–	–	–	4/2	0,12	1	1	–	64	–	32	8	–	–	–	–	–	–	–	-	-	-	>64	4	-	-	-	-	-
17491/2022-1	*V. alginolyticus*	8	–	–	–	–	4/2	0,12	0,5	1	–	64	–	32	8	–	–	–	16/2	–	–	–	-	-	-	>64	8	-	-	-	2	-
19361/2022-1	*V. parahaemolyticus*	4	0,25	–	–	0,5	–	–	1	–	–	-	0,25/4,75	–	8	–	–	2	32/2	2	–	–	-	0,5	-	-	16	-	-	-	-	-
20616/2022-1	*V. parahaemolyticus*	8	0,5	–	–	–	8/4	0,12	2	1	–	>256	0,25/4,75	32	32	–	–	-	16/2	-	–	–	-	-	-	>64	>16	-	-	-	4	-
21249/2022-1	*V. parahaemolyticus*	8	–	–	–	–	–	0,12	1	1	–	>256	–	32	16	–	–	–	–	–	–	–	-	-	-	-	16	-	-	-	2	-
21250/2022-1	*V. parahaemolyticus*	4	–	–	–	–	2/1	-	0,5	–	–	128	–	16	8	–	–	–	–	–	–	–	-	-	-	-	8	-	-	-	2	-
21252/2022-1	*V. parahaemolyticus*	4	–	–	–	–	2/1	0,06	1	–	–	>256	–	32	8	–	–	–	–	–	–	–	-	-	-	32	16	-	-	-	2	-
22158/2022-1	*V. vulnificus*	16	–	–	–	–	–	–	4	–	–	>256	–	–	32	16	–	–	–	–	–	–	-	-	-	-	8	4	-	-	4	-
22226/2022-1	*V. parahaemolyticus*	8	–	–	8	–	–	0,03	2	–	–	>256	–	32	16	–	–	–	–	–	–	–	-	-	-	-	16	-	-	-	2	-
22613/2022-1	*V. cholerae*	16	–	–	–	–	2/1	–	1	–	0,12	>256	–	2	32	–	–	–	–	–	–	–	-	-	1	-	8	2	-	-	-	-
23090/2022-1	*V. parahaemolyticus*	8	–	–	–	–	–	0,12	1	2	–	>256	0,25/4,75	–	16	–	–	–	–	–	–	–	-	-	-	-	16	-	-	-	2	-
23093/2022-1	*V. parahaemolyticus*	8	–	–	–	–	–	0,06	1	1	–	>256	0,25/4,75	–	16	–	–	–	–	–	–	–	-	-	-	-	16	-	-	-	2	-
24626/2022-1	*V. parahaemolyticus*	8	–	–	–	–	4/2	0,03	2	–	–	>256	1/19	32	16	–	–	–	–	–	–	–	-	-	-	>64	16	-	-	-	2	-

24639/2022-1	*V. parahaemolyticus*	8	–	–	–	–	4/2	0,12	2	1	–	>256	0,5/9,5	32	16	–	–	–	–	–	–	–	-	-	-	>64	> 16	-	-	-	2	-
25214/2022-1	*V. parahaemolyticus*	8	–	–	–	–	4/2	0,12	2	–	–	>256	0,25/4,75	32	16	–	–	–	–	–	–	–	-	-	-	-	16	-	-	-	2	-
25215/2022-1	*V. parahaemolyticus*	8	–	–	–	–	2/1	0,06	2	1	–	>256	0,25/4,75	32	16	–	–	–	–	–	–	–	-	-	-	>64	16	-	-	-	2	-

FOX: cefoxitin; AZI: azithromycin; CHL: chloramphenicol; TET: tetracycline; AXO: ceftriaxone; AUG2: amoxicillin/clavulanic acid 2:1 ratio; CIP: ciprofloxacin; GEN: gentamicin; NAL: nalidixic acid; MERO: meropenem; FIS: sulfisoxazole; SXT: trimethoprim/sulfamethoxazole; AMP: ampicillin; STR: streptomycin; AMI: amikacin; P/T4: piperacillin/tazobactam constant 4; TGC: tigecycline; TIM2 ticarcillin/clavulanic acid constant 2; LEVO: levofloxacin; NIT: nitrofurantoin; DOR: doripenem; MIN: minocycline; ETP: ertapenem; IMI: imipenem; PIP: piperacillin; FAZ: cefazolin; TOB: tobramycin; TAZ: ceftazidime; A/S2: ampicillin/sulbactam; AZT: aztreonam; FEP cefepime.
